# Stress Distribution Changes after Root Canal Therapy in Canine Model: A Finite Element Study

**Published:** 2008-10-01

**Authors:** Allahyar Geramy, Mohammad Jafar Eghbal, Sara Ehsani

**Affiliations:** 1 *Department of Orthodontics, Dental School, Dental Research Center, Tehran University of Medical Sciences, Tehran, Iran*; 2 *Department of Endodontics, Iranian Center for Endodontic Research, Dental Research Center, Dental School, Shahid Beheshti University of Medical Sciences, Tehran, Iran*; 3 *Oral Radiologist, Dental Research Center, Shahid Beheshti University of Medical Sciences, Tehran, Iran*

**Keywords:** Finite Element Analysis, Root Canal Therapy, Stress

## Abstract

**INTRODUCTION:** The fracture strength of endodontically treated teeth compared to vital ones has long been a source of controversy. It is not clear how root canal therapy affects the stress distribution in teeth. The purpose of this study was to evaluate the changes in stress distribution after root canal therapy in a human maxillary canine by finite element analysis (FEM).

**MATERIALS AND METHODS:** Two 3D FEM models of a maxillary canine were created; one represented a virgin tooth and the other represented the same tooth after root canal therapy. A single force of 14.1 N was applied 45 degrees to horizontal plane to the center of the palatal surface; stress distribution was then analyzed in both models.

**RESULTS:** SEQV (VonMises stress) analysis demonstrated an obvious decrease after root canal therapy and the regions near cementoenamel junction (CEJ) showed the highest displacement. The endodontically treated tooth demonstrated higher deflection than the vital one.

**CONCLUSION:** Maximum stress and displacement was repeatedly found in the cervical area, hence more emphasis should be placed on the reinforcement of this region.

## INTRODUCTION

A serious concern in restorative dentistry is the biomechanical failure of nonvital teeth (1). As an empirical observation, endodontically treated teeth should preferably be restored with crowns; while even vital teeth with extensive caries can be restored by routine restorative materials. Whether there is a difference in fracture strength between vital and nonvital teeth has long been a subject for debate (2-10).

It has been postulated that the loss of tooth structure from endodontic access and caries removal significantly weakens the tooth (2-4); the greater the loss of tooth tissue specifically dentin the greater susceptibility to fracture (5).

Sedgley found no significant difference between the biomechanical characteristics of endodontically treated teeth and controls, and concluded that root canal therapy does not make teeth more brittle (6). Other studies have concurred with this finding (7) including the one by Steele and Johnson that evaluated the mechanical properties of root treated teeth. They did not find any significant difference in fracture strength after preparation of access cavity (8).

Other studies have argued that root treated dentin does in fact become weak and brittle compared to vital dentin when tested by the unconstrained punch shear test (9). Trope showed a significant weakening of roots after canals instrumentation (10), though another study indicated that endodontic procedures only have a small effect on the tooth (4).

Finite Element Analysis (FEA) is a method to analyze stress distribution in complex structures using their material properties. FEA results in an accurate modeling of complex geometry and analysis of the stress-strain patterns induced within these structures (11).

This method can confirm basic points (12) evaluate theoretic situations (13), as well as normal situations such as tooth movement, (14-15) be a useful indicator in alveolar bone resorption during tooth movement (16-18), in extra oral force application in orthodontics (19) as well as for optimization of orthodontic mechanotherapy (20) or treatment procedure (23), and also for finding answers to clinical questions (21-25).

**Figure 1:**
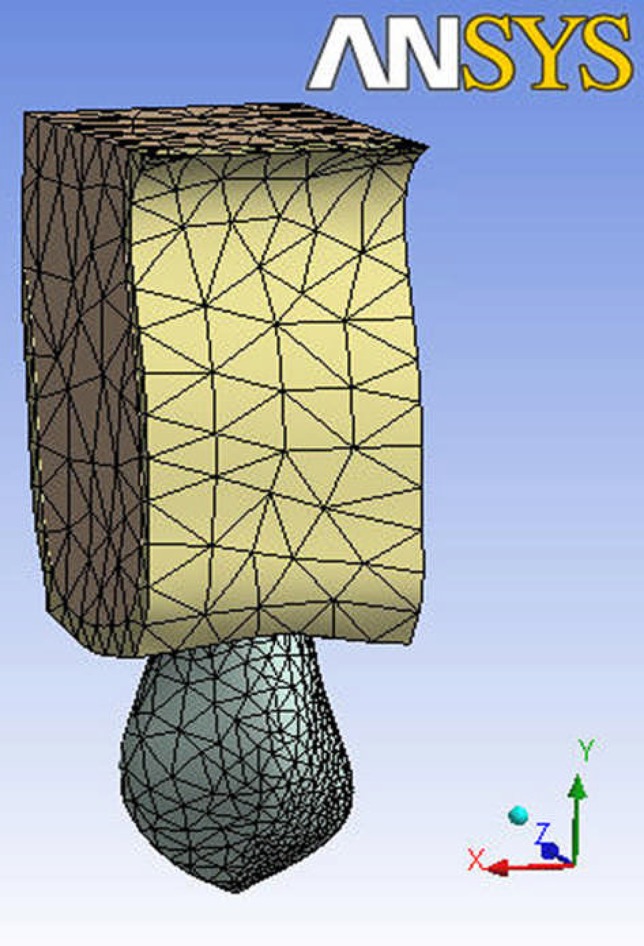
The meshed maxillary canine model

The aim of this study was to evaluate the changes in stress distribution after root canal therapy in a human maxillary canine.

## MATERIALS AND METHODS

**Figure 2:**
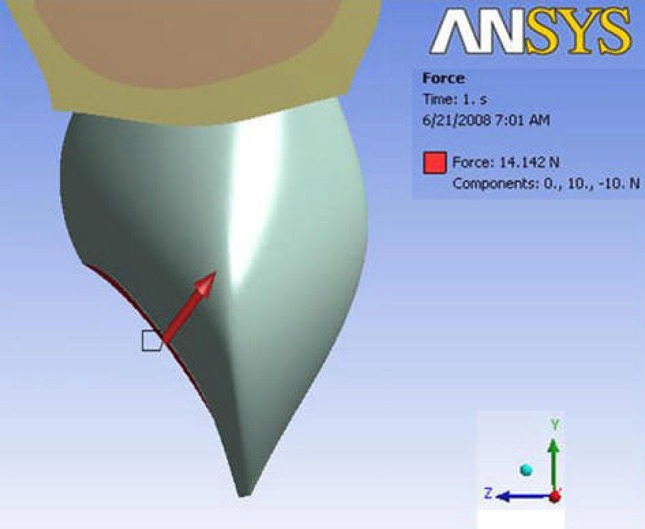
Force application

Two three-dimensional (3D) finite element models of a maxillary canine were created in SolidWork (2006) and then sent to ANSYS Workbench 11.0 (ANSYS Inc. Southpointe, 275 Technology Drive Canonsburg PA 15317, USA). PDL was modeled with uniform thickness of 0.25 mm around the root. The first upper canine model was hence produced with its pulp and crown covered with enamel, the roots were surrounded by PDL and spongy and cortical bone. We decided to model a straight root and canal to eliminate the effects of curvature. In the second model, access cavity was formed, root canal was prepared to a file size #40, the root canal initially had a elliptical opening 4.36×3.2 mm and gutta-percha was simulated to fill the canal and the access cavity was filled by composite. According to previous studies, simulated canine models do not contain cementum being incorporated in the dentin instead. (26). Table 1 shows the material properties considered in this study. All materials were isotropic and homogeneous. The first model presents an intact (vital) tooth consisting of 27112 nodes and 14085 body elements and 6693 contact elements and the other showed the same tooth after root canal therapy (RCT) and consisted of 22527 nodes and 9515 body elements and 5137 contact elements based on Ash dental anatomy (27). A combination of 10 node quadratic tetrahedron, quadratic triangular contact and quadratic triangular target were used in meshing (Figure 1). We applied a single force of 14.1 N 45 degrees to the palatal centre in the horizontal plane (Figure 2).

**Table 1:** Mechanical Properties of used materials

	**Young’s Modulus**	**Poisson’sRatio**
Spongy Bone	13400	0.38
Cortical bone	34000	0.26
PDL	6.67	0.49
Dentin	14700	0.31
Enamel	84100	0.33
Gutta-percha	186	0.25
Composite	6100	0.24

All nodes at the base of the model were fixed to avoid any motion while under load. We defined a path of nodes between incisal and apical region on the most prominent part of the labial surface of the tooth (tooth path).

**Figure 3:**
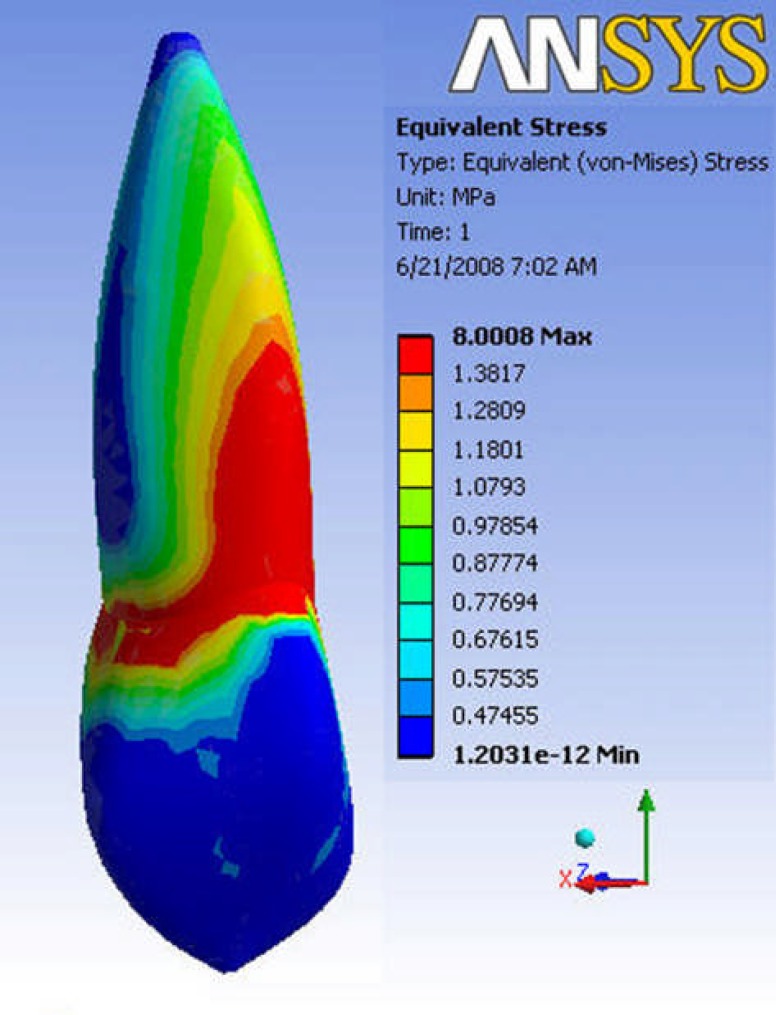
The Pattern of VonMises stress distribution along tooth structure

SEQV (VonMises stress) values of all the nodes along the paths in the models were derived and compared. Displacements were analyzed comparing “tooth path” to a straight line connecting apical and incisal edge of the model. Deflection of the model was assessed by the distances between each curve and their tangential straight line.

## RESULTS

SEQV (VonMises stress) and deflection of the models were calculated and the two modes were compared. “Tooth path” was analyzed, regarding stress distribution and displacement.

**Stress distribution:**

The patterns are almost the same before and after RCT (Figure 3).

**Figure 4:**
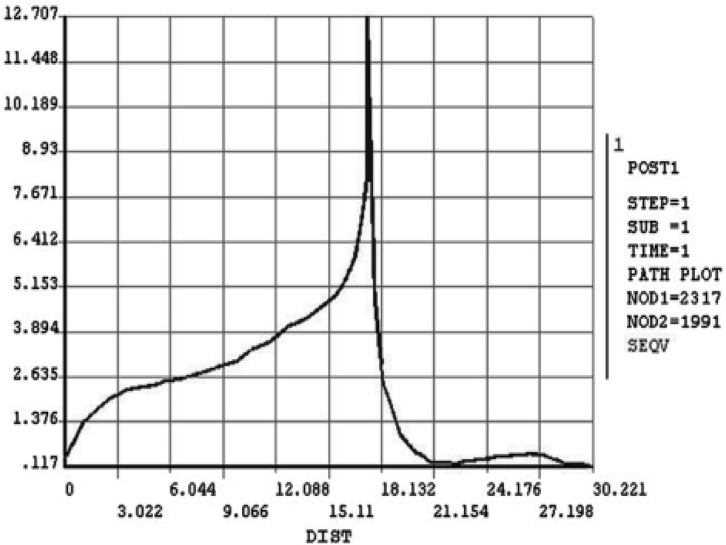
Vonmises stress in the inciso-apical path in a vital tooth

**Figure 5:**
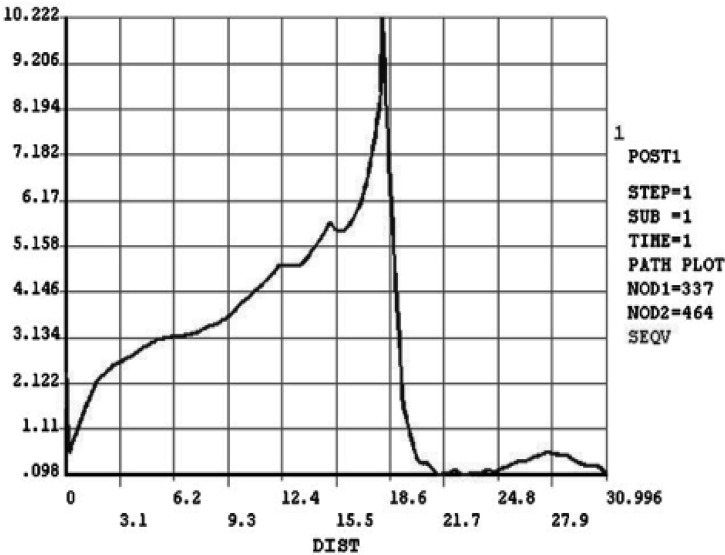
Vonmises stress in the inciso-apical path after RCT

As seen in Figure 4 and Figure 5, there is a gradual increase in stress along the root, reaching a maximum in cemento-enamel junction (CEJ). It then decreases in coronal nodes.

The maximum stress was 12.707 MPa in vital tooth, which decreased to 10.222 MPa after RCT. The lowest stress along tooth path was about 0.12 MPa in the apex and incisal edge in vital tooth. The amount decreased to less than 0.1 MPa after RCT.

**Figure 6:**
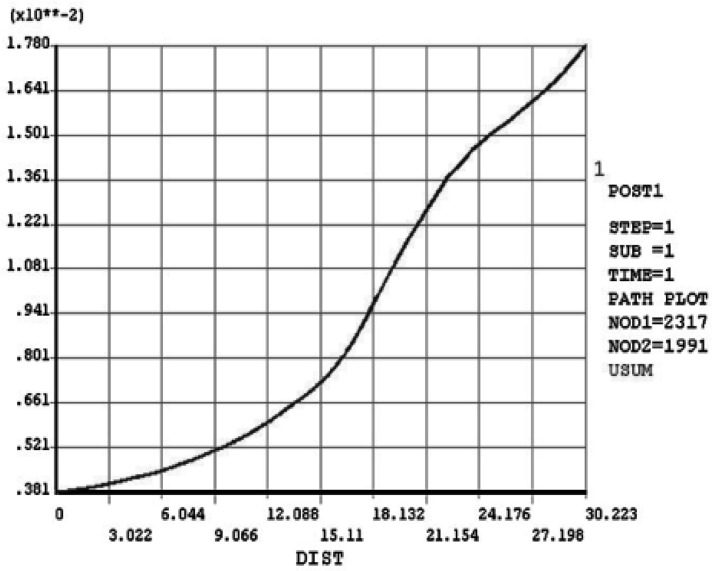
Displacement derived along the “tooth path” in vital tooth

**Figure 7:**
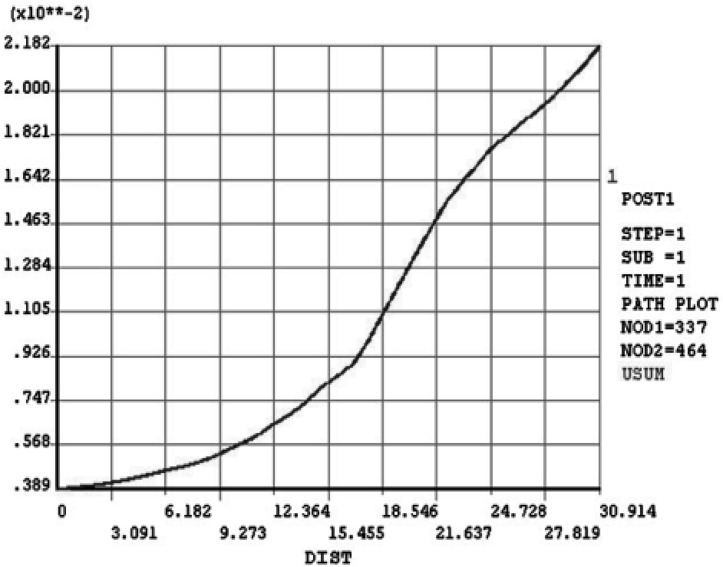
Displacement derived along the “tooth path” after RCT

**Figure 8:**
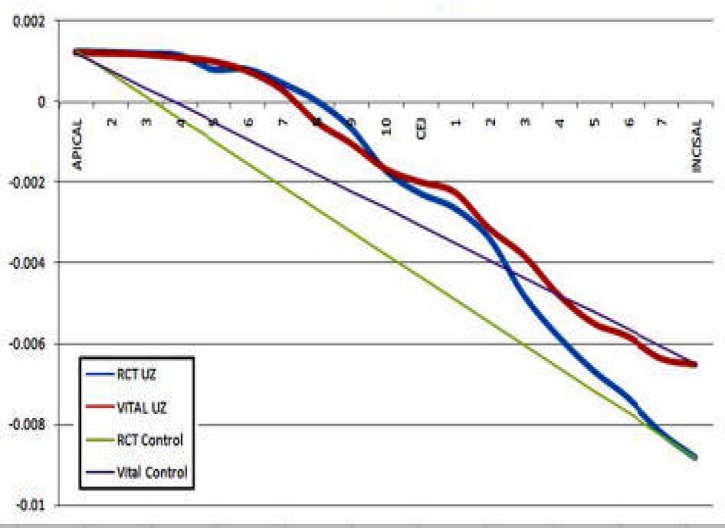
Superimposition of displacements along tooth path before and after root canal therapy

**Displacement**:

Figure 6 and Figure 7 show output data for displacement derived along the “tooth path” before and after RCT. Diagrams show the highest distance near CEJ. In the next diagram the endodontically treated tooth demonstrated a higher deflection than the vital one (Figure 8).

Numeric findings of the nodes along tooth path before and after RCT are shown in Table 2 which shows the difference in CEJ before and after RCT.

The straight lines in Figure 8 could be considered as the movements of rigid teeth without any deflection. Distance between the curve and the straight line shows the flexion of tooth; being higher in the RCT model.

The maximum amount of displacement in vital tooth is 1.78×10^-2^ mm which occurs in incisal edge and increases to 2.182×10^-2^ mm after RCT under the same load.

## DISCUSSION

This study comprised of a three-dimensional finite element model of a maxillary canine and provided an overall view to stress distribution in an endodontically treated tooth. However the results only represent one canine and are likely to be variable in other teeth.

We selected a straight rooted canine to omit the effects of root curvature as there have been reports that natural teeth may experience greater stress due to their root shape irregularities (28).

Additionally a round canal was considered similar to the shape produced by rotary instruments. Furthermore we had not aimed to analyze stress patterns during filing, being strongly influenced by canal shape. It has also been reported that occlusal loads do not significantly change the stress pattern in canal walls (27).

**Table 2:** Displacements (×1000) along tooth path before and after root canal therapy in millimeter

	RCT	Vital
	displacement e-3	displacement e-3
**APICAL**	1.2412	1.195
2	1.2226	1.175
3	1.1911	1.149
4	1.1421	1.072
5	0.7937	0.986
6	0.7997	0.745
7	0.4624	0.291
8	0.01819	-0.51
9	-0.6519	-1.04
10	-1.7057	-1.68
**CEJ**	-2.2792	-2.01
1	-2.6484	-2.25
2	-3.3945	-3.15
3	-4.8138	-3.82
4	-5.835	-4.79
5	-6.6767	-5.49
6	-7.3455	-5.82
7	-8.2027	-6.36
**INCISAL**	-8.8287	-6.51

The maximum stress was found in the cervical region; whereas Hong reported an increase in stress towards the orifice and coronal 1/3 (29). Ricks-Williamson also, reported the maximum stress between the middle and coronal thirds of the root (3).

This study demonstrated decreased enamel stress after root canal therapy, which can be accompanied by an increased tooth flexure at CEJ. This could be explained by tooth structure loss during access preparation; creating a more flexible enamel.

Our findings were also consistent with Ricks-Williamson’s paper; the enamel demonstrated similar behavior to dentin (3).

This simulation study also indicated that VonMises stress was higher in enamel than in the whole tooth; this was somehow similar to Ricks-Williamson who reported that Vonmises stress was higher in enamel than in dentin (3).

Moreover the entire tooth, displayed lower stress levels after root canal therapy simulation. This is consistent with the explanation above; however Pierrisnard found *more* stress in teeth after root canal therapy yet also reported maximum stress in the cervical region, as we did (30).

In a study on tooth deflections under functional loads, Geramy *et al.* reported the maximum deflections in the CEJ area as that area bears the maximum stress (22).

Rees also showed that peak tensile and shear stresses after cavity preparation in second mandibular premolar, occurs in the buccal cervical region, resulting in non-carious cervical tooth loss (31).

Kishen reported that structure loss makes teeth more prone to fracture after root canal therapy (5); however we found greater displacement after RCT than in vital one.

Sedgely, considered punch shear strength, toughness and load to fracture, showed that there are no differences between vital and endodontically treated teeth (6). Other studies argue teeth are more susceptible to fracture post RCT (4).

## CONCLUSION

The results encourage us to conclude that maximum stress and displacement may occur in the cervical region, indicating greater protection for this region after root canal therapy.
